# Personalized digital pipeline for basal cell carcinoma delineation using hyperspectral imaging

**DOI:** 10.1364/BOE.592641

**Published:** 2026-09-01

**Authors:** Paulina Ibek, Alexander Degener, Magne Stridh, Marcus Tegnér, Patrik Edén, Victor Olariu, Malin Malmsjö, Aboma Merdasa

**Affiliations:** 1 Ophthalmology, Department of Clinical Sciences Lund, Lund University, Skåne University Hospital, Lund, Sweden; 2 Computational Science for Health and Environment, Department of Earth and Environmental Sciences, Lund University, Lund, Sweden

## Abstract

Basal cell carcinoma (BCC) is the most common form of skin cancer with the current diagnostic procedures relying on surgical excisions and histopathological analyses. Incomplete removal of the tumor necessitates additional surgery, which is both costly and time-consuming. To address this, non-invasive diagnostic techniques capable of determining the dimensions of skin cancer preoperatively are urgently needed. This work presents the development of an automated diagnostic pipeline for delineating BCC, using hyperspectral imaging (HSI), combined with machine learning (ML). HSI is a non-invasive, high-resolution imaging technique capable of differentiating tissue structures based on their molecular composition. ML is a branch of AI where algorithms learn patterns from data to make predictions without being explicitly programmed. The proposed pipeline is designed to adapt for individual patients, as universal skin cancer characteristic signals are challenging to define. The pipeline predicted tumor sizes with high accuracy, as validated by histopathological measurements in the 21 samples evaluated. The median absolute errors were low (0.25–0.30 mm), meaning that the predicted tumor dimensions were within the clinically accepted safety margins for radical excisions (≤0.4 mm). This work is the first to use unsupervised ML for tumor delineation in BCC using HSI, which enables personalized preoperative assessment and facilitates surgical precision.

## 1. Introduction

Non-melanotic skin cancer was the fifth most incident type of cancer in 2022, with over 1 200 000 cases globally [1]. Although only ranking 22nd in mortality, these tumors can be locally invasive and cause destruction of tissue. The most common type is basal cell carcinoma (BCC), stemming from mutated basal cells located in the outermost layer of the skin, the epidermis. Commonly, skin tumors are treated through surgical excision whereafter the tissue is analyzed by a pathologist who determines the type, stage, and whether the sample is radical (i.e. the tumor has been removed in its entirety). In the case of non-radicality, the patient is called back for a re-excision, where the process is repeated until the patient is cured. The time for receiving a histopathological answer varies from a couple of days up to weeks, with 80% of diagnostic biopsies being reported within 10 calendar days [2]. To increase the chance of a radical primary excision, a margin of seemingly healthy tissue around the tumor is also removed. This is especially relevant in locations with little excess skin, such as the face and head, and particularly around the eye. Excessive tissue removal in these areas threatens anatomical function and aesthetics [3], requiring additional reconstructive surgeries, such as skin grafts or flaps, which are costly, time-consuming and can decrease the quality of life of the patient [4,5]. There is a clear need for methods that can non-invasively determine the tumor border to help guide surgeons preoperatively, to potentially decrease the number of re-excisions and reconstructive surgeries.

Dermatoscopy is a standard approach for preliminary screening. However, to make a reliable diagnosis, histopathology with chemical staining is needed to generate necessary spectral contrast. Non-invasive imaging techniques, such as hyperspectral or photoacoustic imaging have recently demonstrated applicability for the assessment of skin tumors by providing molecular information of the tissue without the need for biopsy [6–9]. These modalities generate high dimensional data, which requires complex analysis tools. Several studies have demonstrated the potential of HSI for delineating BCCs. However, the correspondence between HSI-based delineations and histopathological dimensions remains insufficiently precise to support clinical implementation [10,11].

Machine learning (ML) has been applied in skin tumor diagnostics, recently summarized by Naqvi et al. [12]. However, most models are trained on manually labeled data, with tumor boundaries delineated by hand [13,14]. These approaches lack clinical relevance as, unlike histopathology, they cannot reliably differentiate healthy from pathological tissue, which is essential for accurate diagnosis. A more suitable approach is to construct an ML method using imaging data rich in spectroscopic information, which can reliably, and non-invasively delineate the tumor based on differences in molecular composition [15,16].

Previous studies have highlighted the challenge of the lack of a universal skin tumor signal, which must be addressed for clinical feasibility [7]. Traditional ML classification tasks, such as distinguishing between tumorous and non-tumorous tissue, rely on training large models with extensive datasets. The goal is to uncover meaningful patterns that enable accurate predictions across many samples [17,18]. However, large variations in skin characteristics between individuals obscure any potential general differences between healthy and diseased tissues, rendering such methods impractical with the limited number of samples available in typical clinical settings. Ideally, a skin tumor delineation algorithm should not be dependent on the results from other samples and instead be tailored to each patient.

Most reports involving HSI and ML used the vast spectral information for tumor classification, which has promising results with important clinical implications [19]. However, there is a lack of studies focusing on accurate delineation of the tumor which fulfills a different part of the clinical process. Classification informs on the tumor margins required according to set guidelines [20], which is currently diagnosed with excisional biopsy and histopathology. Meanwhile, delineation would provide a pre-surgical measure to guide the initial excision. There is a lack of reports detailing how these margins can automatically be determined pre-surgically.

In this work we acquire hyperspectral imaging data from 21 BCC samples immediately after surgery, ex vivo and describe an unsupervised, personalized ML algorithm for the automatic delineation of tumors. The key component of the algorithm is that it is intrinsically tailored to each patient, making it adaptable and independent of other tumors, thus avoiding the need for characterizing a universal skin tumor signal from a large dataset. The intention is that the training of the model should not require input from a medical professional, such as a histopathologist, which minimizes bias. However, the model is extensively validated against histopathological results to ensure clinical applicability.

## 2. Materials & methods

### 2.1. Ethics

The experimental protocol for this study was approved by the Ethics Committee at Lund University, Sweden. The research was performed according to the 2013 Declaration of Helsinki. The participants were given both verbal and written information about the study and its voluntary nature. Written consent was obtained from all participants.

### 2.2. Patient characteristics and histopathological analysis

Included in the study were 19 patients with a total of 21 BCC tumors presented to the clinic of Dermatology at Skåne University Hospital (SUS) with BCC tumors needing surgical excision. Exclusion criteria included the inability to provide informed consent or cooperate during the data gathering procedure due to physical or cognitive conditions. 11 tumors were classified as BCC type 1a and 1b according to the Swedish Sabbatsberg model, corresponding to low-risk tumors according to international classification, while 10 tumors were high risk BCC type 2. No metatypical features were present. All tumors were radically excised. The group included 12 women and 7 men with dominantly fair skin of Fitzpatrick class 1-2 with one patient having class 3. The average age was of 72 (range of 35–87) years. The largest tumor diameter was measured in a histological section, routinely stained with hematoxylin and eosin and scanned using the program SECTRA. An important source of uncertainty is formalin fixation; the sample shrinks slightly during fixation in formalin. This means that the ex vivo measurement may be affected after fixation and the subsequent dehydration process.

### 2.3. Sample preparation and histopathological examination

The tumors were excised under local anesthesia at the Department of Dermatology, placed in an isotonic saline solution and transported to a laboratory next door. Any hair was cut off from the samples before scanning. The sample was photographed and then scanned with HSI. The samples were then placed in formaldehyde and sent for histopathological examination. The BCC tumor cross-sections were examined and delineated by a professional histopathologist, without knowledge of the results from the HSI analysis.

### 2.4. Hyperspectral imaging

**Fig. 1.**
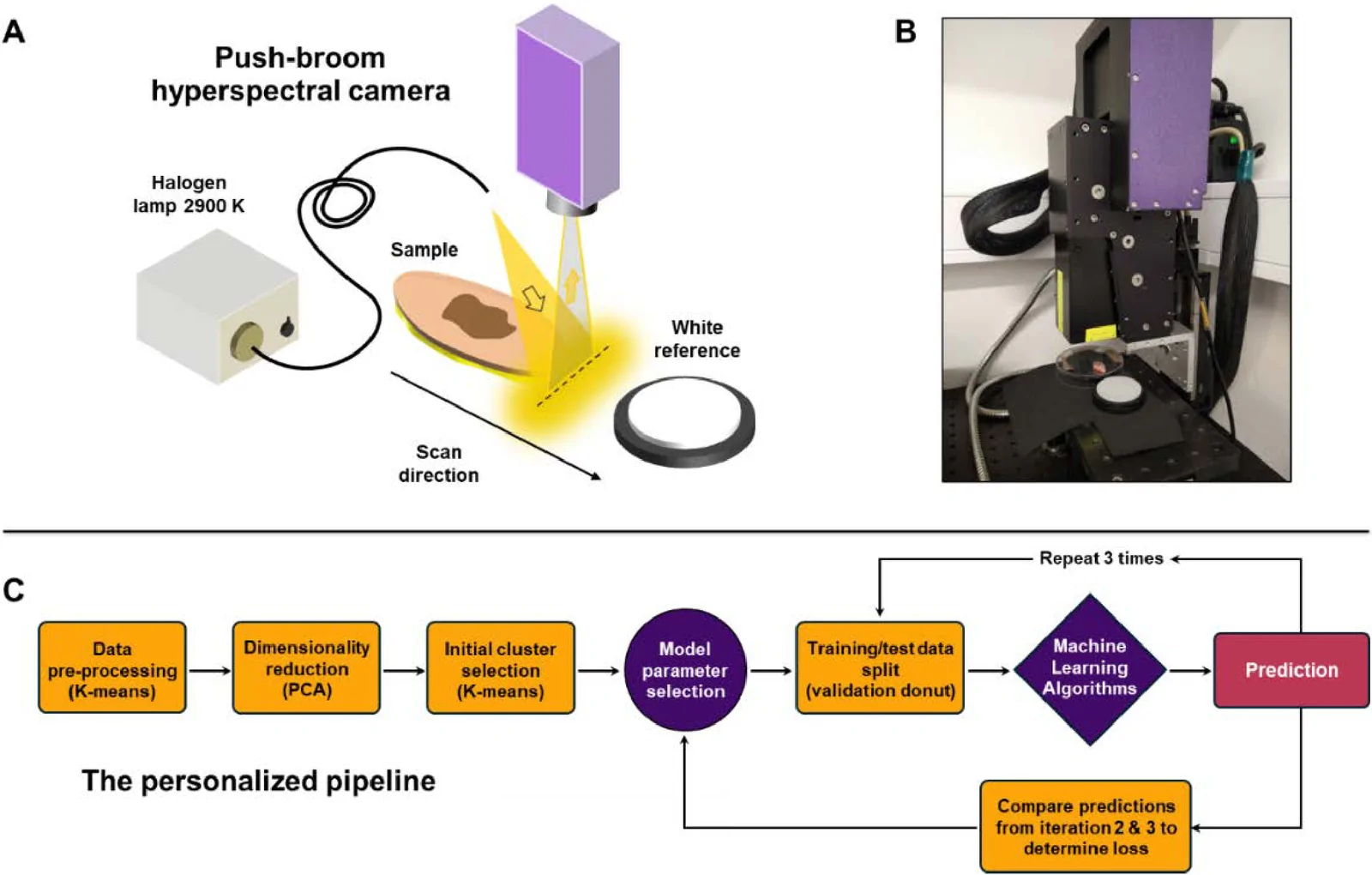
**HSI Method & Overview of diagnostic pipeline.** (A) Schematic of the push-broom hyperspectral camera including the halogen/tungsten lamp operating at 2900 K. The illumination and detection are fixed relative to one another and scan across a surface where the sample is placed followed by a white reference. (B) Photo of the system prior to acquisition of a tumor sample. (C) Flow chart showing the overall progression of the diagnostic pipeline. The data are preprocessed with K-means and reduced with principal component analysis (PCA). Clustering is again applied using K-means to extract a preliminary tumor prediction. Model parameters which regulate the split of the data into training and test sets are selected, and a binary classifier is used to predict the border of the tumor. The result is used to evaluate the model parameters, which are updated, to produce a new prediction.

HSI operates by illuminating the tissue using a broad-spectrum lamp, which is safe for the skin, and capturing diffusely reflected light across hundreds of spectral bands. To acquire detailed spectral information on a surface, the spatial dimensions must be reduced from 2 to 1. Therefore, the instrument scans the tissue line by line to get that dimension back. This technique provides a more comprehensive understanding of the molecular makeup of the tissue. It also enables detailed analysis of components in the tissue, which influence light absorption and reflection. These spectral signatures allow HSI to differentiate tissue types and detect abnormalities. The hyperspectral imaging setup used in this study involved a custom-built HySpex model series camera (Norsk Elektro Optikk AS, Oslo, Norway), designed for spatial scanning with high spectral and spatial resolution. The spectral sensitivity is 400–1700nm, with 382 channels, a spatial resolution of 150 µm and with a spectral resolution of 3.4 nm. A tungsten-halogen lamp operating at 2900 K was used to illuminate the tissue sample, providing broad-spectrum white light with an emission peak in the near infrared spectral range (Fig. 1(A)). A more detailed description of the system is found in a previous report [21]. Figure 1(A) shows the push-broom hyperspectral microscope and the acquisition geometry in relation to the lesion and white reference with a picture of the system in Fig. 1(B).

The typical area imaged to capture the entire lesion was around 100 cm^2^, which produced data sets with approximately 105 pixels, each having 382 spectral channels. With this abundance of data, we developed a machine learning pipeline with one of the conceptual aims being that it self-adapts based on the spectroscopic signals acquired from the sample. This is in essence what makes the pipeline personalized since it is in effect one fixed algorithm that generates different hyperparameters governed by the hyperspectral data. For clarity, anytime we describe parameters in the algorithm being set, this is part of the fixed algorithm that then remains consistent for all samples. An overview of the pipeline is provided in Fig. 1(C), with a detailed description of each step in the following sections

### 2.5. Data pre-processing

The sample is separated from the background using K-means clustering algorithm where we set the number of clusters to be 2. The classification of image border pixels is used to assign labels to sample and non-sample clusters. We note that this step will not be necessary in vivo, since all the acquired data will be from biological tissue.

To prepare the data for Principal Component Analysis (PCA), the 3D hyperspectral data cube is reshaped into a list of pixel spectra, where each pixel is represented by 382 intensity values for each wavelength. The coordinates of each pixel spectrum are also recorded for the purpose of creating images that display the analyzed results at various stages of the pipeline. PCA is then applied to this collection of spectra to reduce the dimensionality of the spectral information. The pipeline initially removes all but the first 50 principal components (PCs).

Unlike conventional procedures, the data was not normalized using white reference normalization. White reference measurements are generally necessary to properly normalize the signal. This is important when comparing signals between measurements and it typically requires additional white reference measurements, which may not always be possible on some parts of the body, such as around the eyes where BCC tumors are common. Since the goal is to detect spectral differences between healthy and diseased tissue, rather than to establish a general spectral signature with universal validity for each, we here explore the possibility of omitting the normalization step and using the raw detected signal in the analysis, which should improve clinical applicability. We still included a calibrated white reference (99% Spectralon, OceanOptics, WS-1-SL) in the measurements for completeness and comparison, however, these pixel-spectra were not used in the pipeline analysis to obtain the tumor borders.

### 2.6. Dimensionality reduction with PCA

PCA was applied to the pre-processed hyperspectral data matrix (**X**), producing score (**T**) and loading (**P**) matrices according to **X** = TP* + **E**, including also a residual error matrix (**E**). After performing PCA, the scores are reshaped back into an image stack of same spatial dimensions as the original hyperspectral image data, where now the spectral dimension has been reduced to the first 50 PCs. It was noted that the first PC accounted for 90% of the explained variance, while mostly corresponding to the white reference spectrum. We therefore make an approximate assumption that post-processing normalization can effectively be performed by downweighing PC1 and focusing the analysis on the remaining principal components. We normalize the scores of the retained PCs to give the components equal variance to reduce the dominance of PC1. By boosting all components but the first one, a greater relative weight is given to components where tumor-related spectral signals are contained to a varying extent. We note that the first component is not removed because it does not completely correspond to the white reference and might still contain some information relevant for the detection of the tumor borders.

**Fig. 2.**
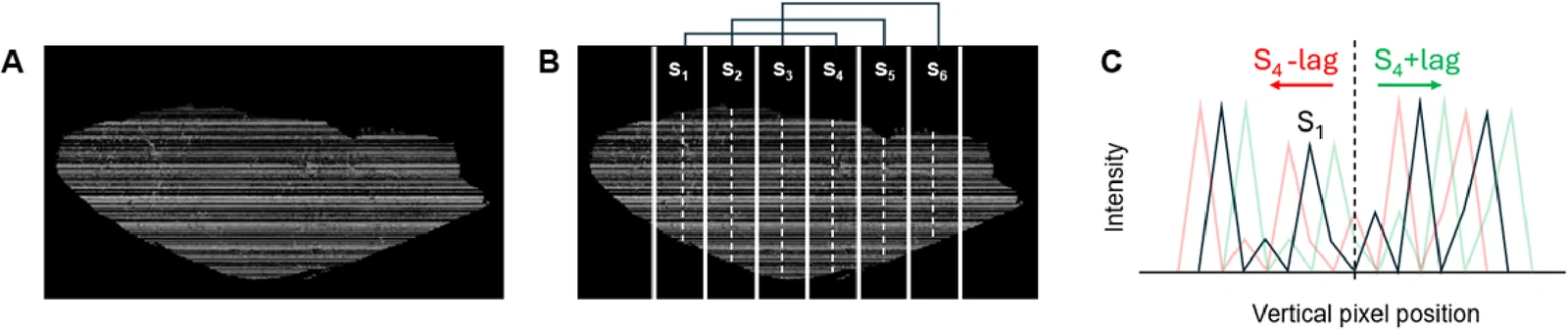
**Identification of principal components with artifacts**. (A) An example of a principal component containing stripe-like artifacts. These components should be discarded before conducting further analysis. (B) The cross correlation is calculated between 3 different section pairs in the sample. For principal components with stripes, the cross correlation between the section will be high when they are aligned (lag zero) and lower once we shift them against each other (lags other than zero). When stripes are not present, this pattern will not be seen. (C) Example of a signal in section S4, showing the spatial shift for positive and negative lags with respect to the vertical intensity profile in S1 that is fixed.

At higher PC index, stripe-like artefacts produced by the instrument (Fig. 2(A)), as well as noise, become dominant, requiring a threshold on the maximum number of PCs to be set to maintain a high signal-to-noise ratio in the data. The artifacts start appearing for varying numbers of PCs for different patients, meaning that the appropriate number of PCs that should be kept varies between patients. This requires an individualized algorithm for their detection.

The presence of stripe-like artifacts is identified by calculating cross-correlation between different pixel section pairs in the direction of the sample perpendicular to the stripes. Initially, the central part of the image is divided into six sections (S1-6) from which the intensity is extracted along the vertical axis at the center of the section (Fig. 2(B)). The intensity cross-correlation is examined between three separate pairs (S1 vs. S4, S2 vs. S5 and S3 vs. S6) by introducing a positive or negative spatial lag between the two intensity profiles (see Fig. 2(C)).

In the presence of stripes, a distinct difference in the cross-correlation should be observed when offsetting the two profiles from their original position in either vertical direction. By extracting the cross-correlation for all multiple spatial lags in each direction from the original location, the appearance of a clear single peak centered at lag = 0 is indicative of the presence of stripes. We note that a high cross-correlation could still be observed in the absence of stripes if the underlying intensity profile lacks distinct features. For this reason, we perform the correlation across the six segments as indicated in Fig. 2(B).

From all the cross-correlations performed for one sample we extract a single average value, the relative cross-correlation, defined as the cross-correlation at lag 0 divided by the mean of cross-correlations at all other lags, which is evaluated against a set threshold to determine the cut-off for discarding PCs. The threshold itself is also determined from the data and obtained by averaging the relative cross-correlation acquired from images produced by PCs 10-25, which consistently is represented by noise in all data sets. For PCs carrying information, the relative cross-correlation was around 0.2 while for PCs dominated by stripe artifacts, a value of ∼0.7 to 0.9 was found. This approach ensures a consistent approach for data truncation that can adapt to each sample.

### 2.7. Tumor identification with validation donut

The reduced data is further clustered using K-means clustering algorithm to separate tumor and non-tumor regions. The clustering is performed on the reduced spectral dimension, without considering any spatial information. However, instead of separating the data into two clusters (i.e. tumor vs healthy tissue), we recognize that both healthy tissue and tumor likely have many different characteristics each, requiring division of the data into more clusters to account for this. Determining the number of clusters can be done in several ways. We here develop an automated approach to remain consistent from sample to sample. For each sample, we let the algorithm perform multiple clusterings, and calculate the silhouette coefficient [22] for each, which is a commonly used metric for evaluating the quality of clustering. The number of clusters with the highest silhouette coefficient is selected automatically. It is important to note that the optimal number of clusters varies across samples and is calculated for each patient. Finding the optimal silhouette coefficient results in a varying number of clusters in the different samples, thus accounting for expected spectral variability between patients and tumors.

Next, the cluster closest to the center is labeled as tumor, and the remaining clusters are grouped together and labeled as non-tumor. This labeling serves as a preliminary step for the ML algorithm. To address misclassifications of regions in the clustering results, we introduce what we call a validation donut to improve the reliability of the training data. Two concentric circles with different radii are placed around the tumor cluster, effectively creating two separated regions representing tumor and non-tumor regions. The inner and outer radii of the validation donut are sample-specific, determined by

$$r_{inner}\;=\;p_{i}\sqrt{\text{std}{\left(x_{tumor}\right)}^{2}+\text{std}{\left(y_{tumor}\right)}^{2}},$$

and

$$r_{outer}=p_{o}\sqrt{\text{std}{\left(x_{non-tumor}\;\right)}^{2}+\text{std}{\left(y_{non-tumor}\right)}^{2}},$$

where *x* and *y* are the positions in x- and y-direction for all pixels in the specified class (preliminary tumor and non-tumor), and *p_i_* and *p_o_* are tunable parameters. The parameters and classification threshold are optimized for each sample during an iterative ML step through 50 iterations of a random search algorithm. The ranges for *p_i_*, *p_o_,* and the threshold are [0.7, 0.9], [0.6, 0.9] and [0.2, 0.4], respectively. The ranges were chosen empirically.

Any pixel-spectrum within the inner radius of the validation donut can be selected for training as tumor by the algorithm, and any pixel-spectrum outside the outer radius can be selected for training as non-tumor. The pixels within the validation donut itself are set aside for validation, and correspond to a region of higher uncertainty, where the initial tumor clustering is deemed inadequate. The final training pixels are selected probabilistically to have balanced classes.

Given these labels, ML models can be trained to classify the remaining pixels. The models are trained on the reduced spectral data only, and do not take into consideration the classifications of neighboring pixels. We apply three ML models (random forest - RF, support vector machines – SVM, and multilayer perceptron - MLP) separately to identify whether differences in prediction accuracy occur. To retain spatial consistency, a uniform convolution kernel of 5 × 5 pixels is applied to the prediction map, where the probability output from each ML model for every pixel is smoothed, yielding a final prediction of the tumor borders.

### 2.8. Model parameter optimization

Three parameters have been identified to have a large effect on the final prediction map: the two tuning parameters determining the inner and outer radii of the validation donut and the probability threshold from the ML models determining the classification of a pixel into either category. These model parameters must be selected carefully to not underestimate nor overestimate the borders of the tumor. The selection of model parameters is best illustrated through an example. A tumor prediction obtained with a specific set of model parameters is treated as the initial tumor cluster and fed back into the ML model. This process generates a new prediction map. If this step is repeated iteratively, an unsuitable set of parameters could cause the predicted tumor to either grow or shrink with each iteration, indicating that the initial prediction was an overestimation or underestimation of the tumor. To identify the optimal model parameters, the ML model is iteratively applied to successive predictions, selecting the parameter set that minimizes the difference between consecutive iterations. The difference is measured between iterations 2 and 3.

The optimal model parameters are used for the final probability map, where the output from the third iteration is used for the final prediction of tumor borders. The ML classification is iterated 3 times for each set of model parameters. A search was conducted to find the optimal ML hyperparameters for only a selected number of samples, due to computational cost. It was found that hyperparameter tuning was not necessary, as it did not have a large effect on the final prediction. The hyperparameters evaluated for the three ML models are summarized in Table 1, with the parameters used in the pipeline in bold. These were then applied to all samples. There was no significant difference in performance for the different hyperparameters. The remaining parameters were default from the scikit-learn library v.1.6.0 for all three models.

**Table 1.** Hyperparameters for the different ML models

ML model	Parameters	Values
MLP	Architecture	**50 nodes**, 100 nodes, 2 layers with 50 nodes each
MLP	Regularization	0.0001, **0.001**, 0.01
SVM	γ	0.01, **0.1**, 1
SVM	C	100, 1000, **10000**
RF	Number of estimators	50, **100**, 200
RF	Max tree depth	**None**, 10, 20

## 3. Results

### 3.1. Data preprocessing and clustering

**Fig. 3.**
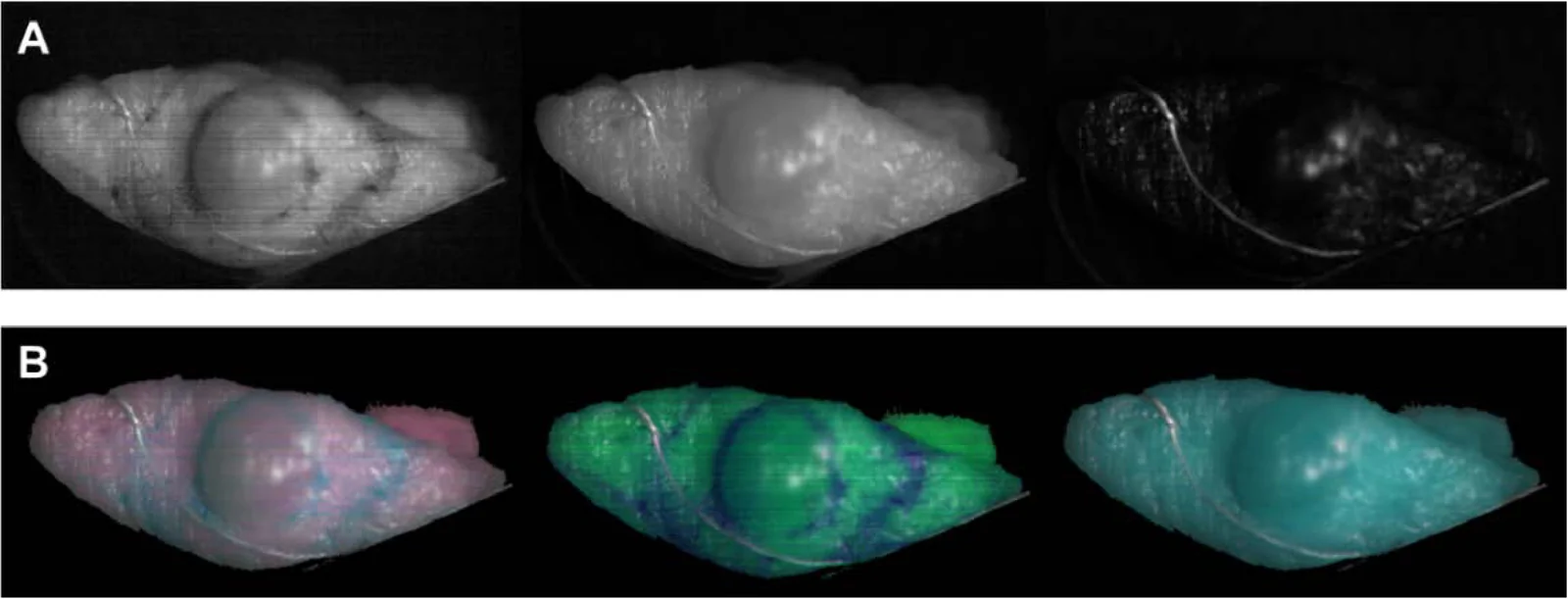
**Unprocessed monochromatic and false color images acquired from HSI.** (A) Monochromatic images extracted from the hyperspectral data cube at three randomly selected spectral channels out of the 382 acquired by the camera, demonstrating the wavelength-dependent tissue contrast. (B) False-color images where three randomly selected channels are mapped to the RGB channels, revealing structural and spectral variations within the tumor tissue.

Figure 3(A) shows three monochromatic images of a BCC tumor surrounded by healthy tissue extracted from the hyperspectral data cube without any pre-processing. The wavelengths were selected at random from the 382 channels to demonstrate that different features appear in different spectral channels. Figure 3(B) displays false color images, each combining three randomly chosen channels each from the 382 available. The images demonstrate the abundance of spectral data that is contained in the measurement where different features become visible depending on wavelength, which calls for a systematic analysis approach.

**Fig. 4.**
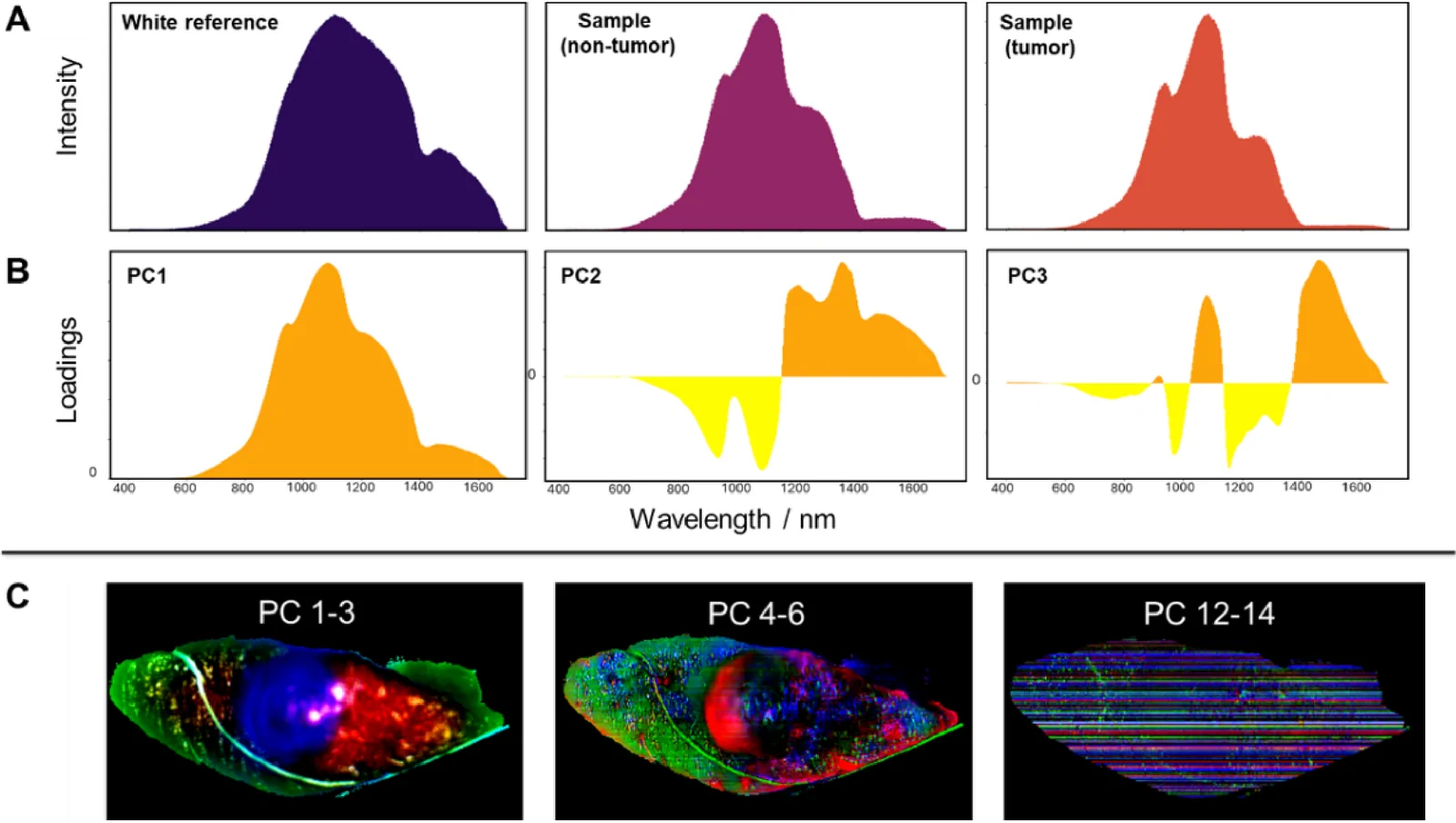
**Spectra from the sample, and principal components highlighting different features.** (A) Spectra from the white reference, non-tumor part of the sample, as well as the tumor part of the sample. We see that although there are some differences in the spectra, the overall shape is similar, due to the data not being normalized. (B) The principal component (PC) eigenvectors showing the wavelength contributions to the first six PCs. (C) False-color images generated by mapping groups of three consecutive principal components (PC1–3, PC4–6, and PC12–14) to RGB channels. Lower-order PCs reveal structural and spectral differences corresponding to tissue composition and tumor regions, while higher-order PCs display characteristic scanning artifacts and striping patterns.

Figure 4(A) shows the detected raw spectra acquired from the white reference and the sample, separating non-tumor and tumor regions. The data is not normalized and it becomes evident the spectral shapes of the sample regions only slightly deviate from the incoming illumination via the reference, which is because only a small fraction of the diffusely backscattered light is absorbed by the tissue. In other words, the excitation spectrum is largely contained in the measured data.

We apply PCA according to the description provided in section 2.6 and plot the loadings from the first three PCs as a function of wavelength (Fig. 4(B)). Here we observe the similarity between the white reference spectrum and the wavelength dependent loadings for PC1, which accounts for 90% of the explained variance of the data. Here it also becomes evident that PC1 contains some similarities to both sample spectra from Fig. 4(A). The loadings of PC2 and PC3, however, differ significantly in character from the first component, confirming that other features are captured.

Figure 4(C) shows an additional set of false color images, which are now constructed from the loadings from three PCs instead of three spectral channels from the RAW data as shown in Fig. 2(B). What now emerges are strong contrasts between different regions of the sample that could not be observed before, which now are likely associated with tissue of different molecular composition, demonstrating the impact of PCA in the pipeline to enhance the spectral contrast. After analyzing all tumors and applying the automatic cut-off threshold as described in section 2.6, we found that all samples could be well characterized by only 4-11 PCs, which is demonstrated by the last false color PC image.

### 3.2. Clustering, training data generation & initial tumor border prediction

**Fig. 5.**
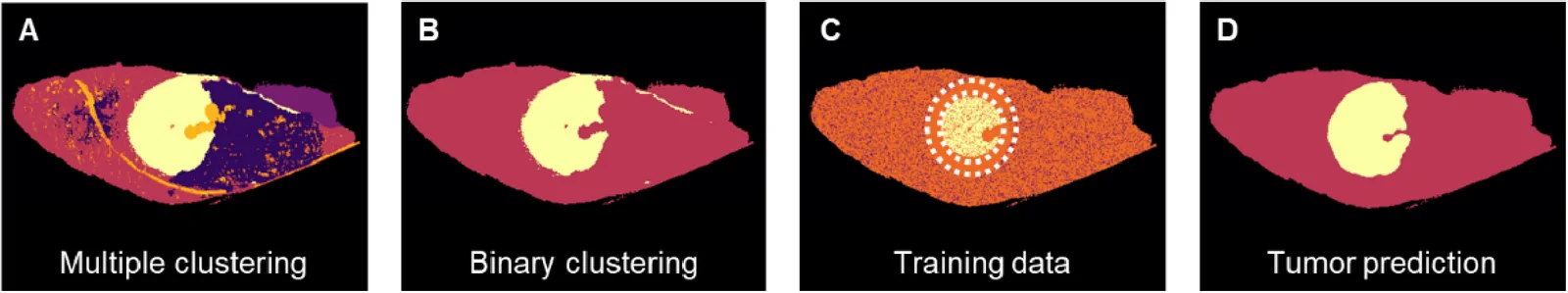
**Illustration of the steps in the proposed machine learning (ML) pipeline for automated tumor delineation.** (A) Clustering – the hyperspectral image is clustered into multiple groups using K-means. (B) Preliminary tumor cluster – the cluster closest to the center of the sample is identified as the initial tumor region. (C) Training data – a validation donut (white dashed lines) defines the regions used for training: pixels inside the inner circle are labeled as tumor, those outside the outer circle as non-tumor, while pixels within the donut are reserved for validation. (D) Tumor prediction – final tumor borders predicted by the ML model after training, showing clear separation between tumorous and non-tumorous tissue.

We apply K-means clustering on the reduced data set as described in section 2.7. In the representative example shown in Fig. 5, the number of clusters was automatically determined to be 5 using the silhouette coefficient and Fig. 5(A) shows how these clusters are mapped out in space. Figure 5(B) shows how the division of the initial 5 clusters have been made into tumor and healthy clusters and how these map out across the sample. Figure 5(C) demonstrates the application of the validation donut that divides all pixels of the sample into three regions where those within the inner circle are labelled tumor and those outside the outer circle are labelled healthy. The middle region is used for validation and once the ML algorithms have been applied the tumor border is predicted (Fig. 5(D)).

### 3.3. Model optimization & refining tumor borders

**Fig. 6.**
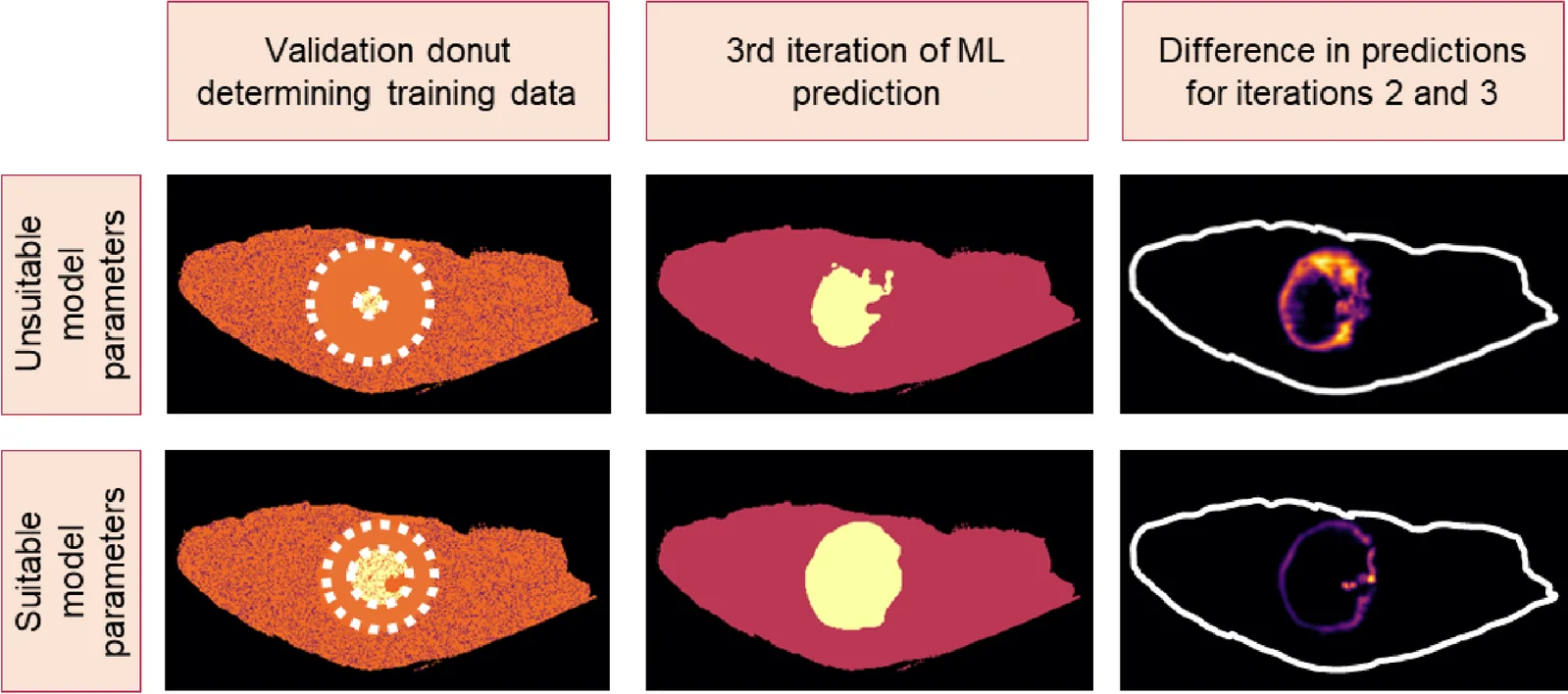
**The selection process of model parameters for an accurate tumor delineation.** Having found preliminary tumor cluster in the sample, a ML model is trained to refine the prediction. A validation donut (white dashed lines) defines the regions used for training, see first column. The inner and outer radii of the validation donut, as well as the threshold for the ML classification, make up the model parameters, which affect the ML prediction. Given a preliminary tumor produced by K-means clustering, a random search is executed to find the most suitable model parameters. The most suitable model parameters are those for which the tumor borders reach a stable state after iterating the ML step three times, see second column. For unfit model parameters, the tumor will either grow or diminish by repeating the ML classification. The stable state is quantified as the difference in tumor probability maps for iteration two and three of the ML step and should be minimized with the best model parameters, see third column. Note that the upper row represents a case where unsuitable model parameters have been chosen, which leads to inaccurately predicted tumor border. The bottom row shows a case where suitable model parameters have been chosen, yielding an accurate prediction.

Once the tumor border has been predicted, the borders are refined using the iterative loop described in section 2.8 in the Methods. This step ensures that the initial prediction is not a result of poorly selected model parameters. By iteratively redefining the model parameters, particularly the radii of the validation donut, based on the returned tumor size, a converging or diverging size difference between iterations report on the stability of the model parameters. The top row in Fig. 6 shows a set of images demonstrating a case where unsuitable model parameters have been used which leads to a diverging difference in size between iterations, and in effect a shrinking tumor. The importance here is not the fact that the tumor shrinks, but rather that the size difference is diverging, which in some cases leads to an iteratively growing tumor. This is placed in contrast to the bottom row where suitable model parameters yield a converging size difference between each iteration, which ultimately leads to the tumor size stabilizing, which lends more confidence to the final prediction.

### 3.3. Prediction vs. histopathology - comparing tumor size

**Fig. 7.**
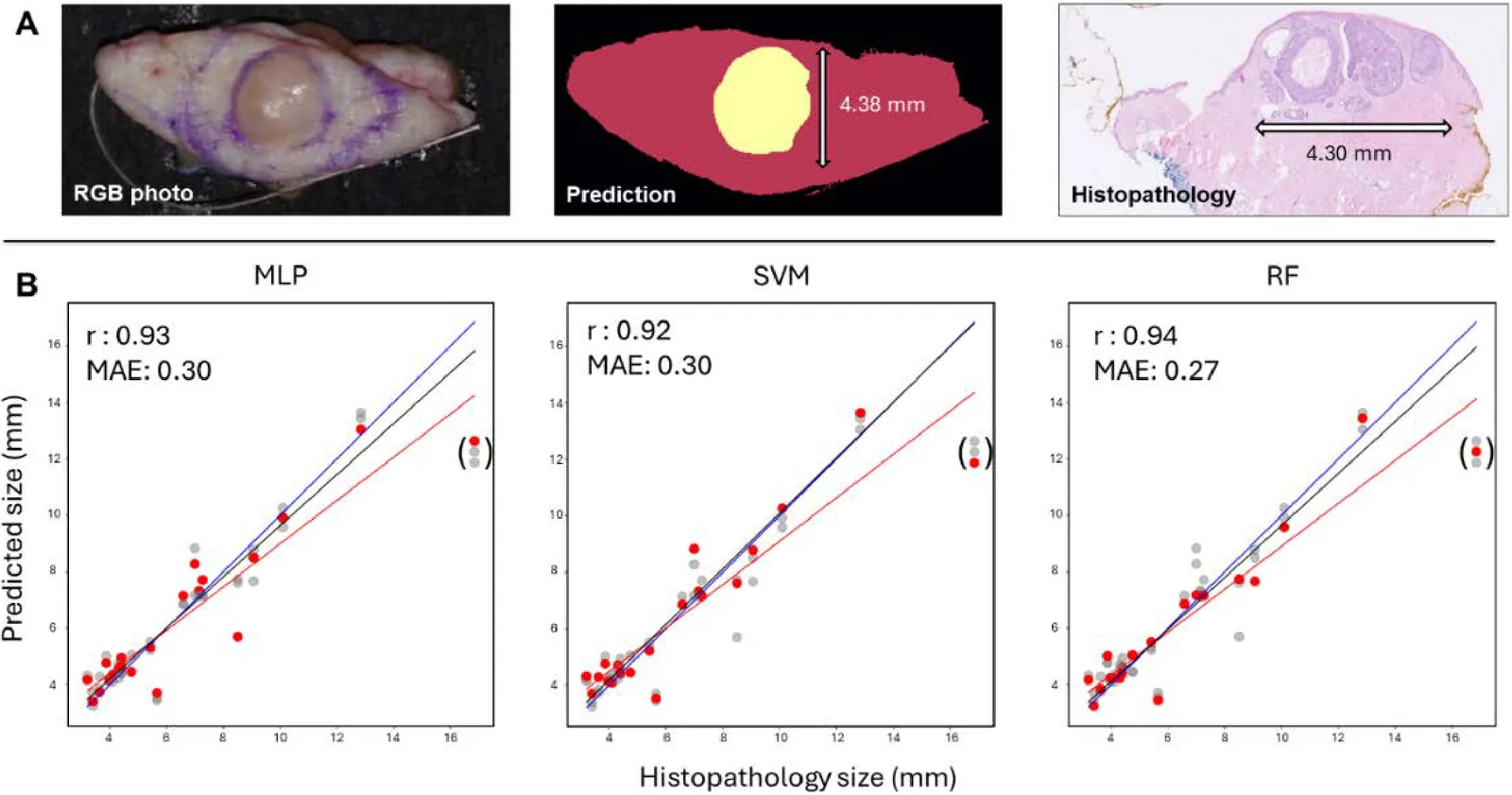
**Comparing results to visual inspection and histopathology** (A) A representative example comparing an RGP photo with the tumor prediction and histopathology obtained at the widest part of the tumor. The pipeline predicts a tumor width of 4.38 mm compared to 4.3 mm obtained with histopathology. (B) Comparison between model predictions and histopathologically determined tumor sizes for all tumors, for three different machine learning (ML) models: multilayer perceptron (MLP) (left), support vector machine (SVM) (middle), and random forest (RF) (right). Each scatter plot shows the predicted tumor size versus the histopathological measurement, with the blue line representing the 1:1 relationship, the red line the regression line including all samples, and the black line the regression line after exclusion of one outlier with a heterogenous and complex appearance (in parentheses). Red points indicate predictions from the model in focus, and grey points represent predictions from the other models for reference. Pearson correlation coefficients (r) and median absolute errors (MAE) are shown for both full datasets (red) and outlier-excluded sets (black). All models demonstrated strong agreement with histopathology (r = 0.92–0.96, MAE = 0.25–0.30 mm), with no significant difference between methods (p > 0.05), confirming the robustness and reproducibility of the proposed hyperspectral–ML pipeline. Note that the ML predictions have a median error which is within the clinically accepted safety margins of radical excisions.

The developed diagnostic pipeline was investigated on a total of 21 BCC samples, and the predicted sizes were compared to tumor widths determined by a histopathologist (Fig. 7). In the histopathological images it is noted in what direction the samples have been cut, but the exact position which determines the size of the tumor is hard to determine, which is discussed further below.

The Pearson correlation for the predictions made by the MLP, SVM and RF models and histopathology were 0.93, 0.92, and 0.94, respectively, demonstrating strong agreement between predicted and histopathological sizes. The median absolute errors (MAE) for the methods were 0.30, 0.30 and 0.27 mm. To determine whether the three methods yielded statistically different results, pairwise t-tests were performed on the residuals of the model predictions. From the analysis it was concluded that there was no difference between the methods at a 5% significance level, indicating that the results are robust across different ML algorithms. Figure 7 shows the predicted and histopathological sizes of the tumors for the three models, with the 1:1 line in blue and regression line in red. The largest tumor in the set, measuring over 16 mm, was identified as an outlier. The model severely underestimates its size, which we attribute to the heterogeneity of the tumor. Removing it from the set and fitting a new regression line (in black), we see considerable improvement, with the new correlation values being 0.93, 0.95, and 0.96 the new median errors 0.29, 0.29, and 0.25 mm. This shows that the pipeline performs particularly well for most tumors, especially those with more homogenous structures. Overall, these findings suggest that the proposed method can already provide reliable estimates of tumor size from spectral data. High predictive accuracy across three independent ML models demonstrates the robustness of the pipeline and shows promise for clinical implementation of our proposed pipeline.

## 4. Discussion

Traditional histopathology remains the gold standard for effective diagnosis and treatment for BCCs. Integrating a non-invasive method for preoperative delineation of tumors into the clinic could help guide surgeons in their excisions, and potentially reduce the risk of non-radical excision, the number of surgeries and waiting time, while keeping tumor margins small. In this context, our study demonstrates how hyperspectral imaging combined with machine learning can provide automated and individualized tumor delineation, highlighting its potential as a clinically valuable complement to existing diagnostic tools.

### 4.1. Clinical relevance & comparison to existing imaging methods

Over the past decades, various imaging modalities such as high-frequency ultrasound, optical coherence tomography, and hyperspectral imaging have been investigated as complementary approaches to help surgeons estimate tumor dimensions and margins. High-frequency ultrasound (HFUS) of BCC have shown a Cohen’s Kappa of 0.8251 between estimated tumor type (e.g. nodular, superficial, infiltrative) and histological results [23]. In a recent study, optical coherence tomography (OCT) demonstrated a Pearson correlation coefficient of 0.83 with histologically determined tumor thickness, with a median difference of 0.12 mm, while HFUS showed a correlation of 0.59 with a median difference of 0.30 mm [24]. In periocular BCC, OCT yielded correlations of 0.80 and 0.66 for horizontal margins in the lateral directions, and a vertical margin correlation of 0.43 with histopathology [25]. HFUS has also been used to assess tumor depth, showing a correlation of 0.97 for localizing deep tumor margins [26]. These studies indicate that imaging-based tumor size estimation can approach the accuracy of histopathology in many cases. The maximum correlation value obtained in the present study (0.96), as well as the lowest median error (0.25 mm), not only fall within this range, but in several cases surpass the performance of conventional imaging techniques, demonstrating that the presented ML pipeline can provide highly accurate, quantitative tumor size predictions. According to Auw-Haedrich et al. [27], the histological margin should be at least 0.2 mm to reduce the risk of recurrence. A margin of 0.5 mm has also been suggested [28], particularly for patients with additional risk factors such as aggressive growth patterns, large tumors, or recurrence. According Quazi et al. [29], guideline margin is 0.4 mm, which is higher than the median absolute error obtained with our pipeline. Importantly, our method achieves these results in a fully automated manner, potentially reducing observer variability and enabling rapid assessment. These findings highlight the promise of ML approaches for improving clinical workflow and enhancing precision in BCC evaluation.

### 4.2. Strengths of the personalized unsupervised pipeline

It is important to note that tumors are inherently heterogenous, meaning a single sample could contain regions resembling different BCC subtypes, such as nodular (type 1A), superficial (type 1B) and infiltrative (type 2). Heterogeneity poses a challenge for unsupervised ML-based analysis, as the model must recognize diverse growth patterns as belonging to one tumor class. As our pipeline identifies several clusters, it provides the foundation for a more detailed study to understand whether these represent real anatomical features, which is beyond the scope of the current study. Nonetheless, the algorithm performed well for 20 of the 21 samples, indicating that it was able to manage variations in tumor characteristics in sample, and capture spectral differences between healthy and tumorous tissue.

A potential concern is whether the proposed validation donut structure introduces a bias toward circular tumor shapes, and how the method performs for irregular geometries. In our dataset, which includes BCC tumor types 1 and 2, we do observe some degree of shape irregularity, and the algorithm performs well in predicting tumor size across these cases. Importantly, the purpose of the donut structure is not to impose a geometric constraint on the tumor boundary, but rather to separate tumor and surrounding healthy tissue. The final tumor delineation is determined by the classifier, which does not enforce circular boundaries. Nevertheless, for highly irregular or elongated tumors that are underrepresented in the training data, performance may be affected. In such cases, the framework could be modified by adapting the validation region to better capture complex geometries, either using ellipses or using a shape defined by the initial clustering step. Future work should systematically evaluate performance across a wider range of tumor shapes to further assess robustness.

More recently, ML has shown promise for segmentation tasks, including applications in skin tumors. Numerous studies have explored how ML can segment tumors; however, most approaches primarily rely on large, annotated datasets and manual delineation by histopathologists. For instance, Wang et al. developed a deep learning-based semantic segmentation method for whole slide images for non-melanocytic skin tumors, achieving high accuracy but requiring extensive manual annotations [30]. Similarly, Thomas et al. provided a dataset of 290 histopathology tissue sections for non-melanoma skin cancers with expert delineations to enable ML-based segmentation [31]. Aloupogianni et al. manually traced the borders of pigmented skin lesions in histopathology images for the training of ML models aimed at segmenting tumors in HSI [32]. In contrast, our study is the first to introduce a ML pipeline tailored to each patient, utilizing HSI to delineate BCC tumors ex vivo, without the need for manual annotations, with proper histological comparison. This approach not only reduces dependency on large, annotated datasets but also offers a personalized diagnostic tool that can be integrated into clinical workflows with minimal manual intervention.

### 4.3. Methodological limitations and sources of uncertainty

While the presented pipeline demonstrates strong performance and clinical potential, there remain opportunities for refinement. The pipeline consists of several steps where critical decisions are made, where adjustments could potentially enhance accuracy and robustness. The first critical decision in the pipeline is reducing the number of principal components. When selecting the components, we do not want to make assumptions about the location of the tumor signal; instead, the designed algorithm only discards the spectral components which are deemed too noisy, since tumor characteristics are unknown. It was discovered that the tumor signal was usually confined to a small number of principal components. However, the specific components most relevant to the tumor varied between samples. This indicates that there are other signals obscuring the tumor, making the tumor identification more challenging. The step which was deemed the most important for an accurate tumor prediction was the selection of the number of clusters. Since tumors are not homogenous masses [33], having too many clusters risks dividing the tumor into multiple sections. This, in turn, would train the ML model only on a subsection of the tumor, leading to an underestimation of the actual tumor border. On the contrary, having too few clusters also led to suboptimal results, as the algorithm in those cases fails to properly separate tumor and healthy tissue.

Potential opportunities for improvement for the selection of number of clusters could be utilizing an alternative metric to the silhouette coefficient. This is because the silhouette coefficient can be misleading for clusters with irregular shapes, outliers or in high-dimensional data. Another approach could entail selecting the principal components more carefully in the previous step, so that only components containing tumor signals are utilized. This would facilitate more optimized clustering since there would be less irrelevant information, and thus a smaller probability of clustering on noise. The automation of selecting relevant principal components would pose a challenge in this scenario, since the algorithm would have to be designed to identify tumor-like signals. Selecting the middle cluster as the preliminary tumor could also be revised. However, the method works well ex vivo due to how excisions are made, and the method is still relevant in vivo with the right positioning of the camera, since it is not meant to be used for screening.

There was no significant difference in predicted tumor size between the three ML models. Changing the ML hyperparameters also had a small effect on the final prediction, especially compared to the preceding steps. Since ML models rely heavily on well-labeled input data, the pre-processing stage, which leads to the labels of tumors/non-tumors, plays a more critical role. Inaccurate or inconsistent annotations cap the maximum achievable performance of any model, regardless of complexity. High-quality labels provide the ground truth that guides the learning process. Therefore, ensuring accurate and robust pre-processing can be more critical than the ML model itself or fine-tuning model architecture and hyperparameters [34,35].

### 4.4. Histopathological reference and measurement uncertainty

In the present study, histopathological measurements were used as a reference standard for tumor size, although these measurements are themselves subject to sources of variability. It is therefore important to note that hyperspectral imaging was performed immediately after excision, before fixation, while histological measurements were obtained only after routine tissue processing, which involves several steps that affect specimen dimensions. These differences should be considered when interpreting the correlation between the two methods and whether systematic offsets could be addressed in the future.

Surgically excised biopsies are known to shrink once placed in formalin due to the elasticity of skin and mechanical relaxation after resection [36–38]. Studies have shown that the largest contraction occurs immediately after excision, although additional contractions can occur during formalin fixation, dehydration and paraffin embedding [39,40]. Moreover, the extent of the contraction is subject to tissue composition, age, and other potential health conditions, making it difficult applying a uniform correction factor when comparing the tumor width obtained with our pipeline and histopathology.

Another source of uncertainty is the correspondence between histological sectioning and the direction in which tumor width is assessed in the hyperspectral images. Histological slices are typically made perpendicular to the long axis of the specimen. To reduce uncertainty related to orientation, we aligned the scan direction of the hyperspectral acquisition with the long axis of the specimen. This means that the vertical direction in the hyperspectral image corresponds approximately to the direction of histological sectioning. Nevertheless, exact spatial matching between the histological section and the region assessed in the hyperspectral image was not possible, which may have contributed to discrepancies between the two measurements.

### 4.5. Future directions and clinical translation

To move closer to clinical application, the method would need to be implemented in vivo, enabling clear visualization of tumor borders and providing guidance prior to excision. Expanding the approach to include additional tumor types, such as squamous cell carcinoma and malignant melanoma, would further increase its utility. Integrating complementary imaging modalities, for example photoacoustic imaging (PAI), could provide additional depth information to improve delineation (15). Nonetheless, even at its current ex vivo stage, the algorithm demonstrates potential for practical use in assisting tumor assessment and surgical planning. A concrete example is Mohs micrographic surgery, which is considered the gold standard treatment for high-risk BCCs and other skin tumors in esthetically sensitive locations such as the head and face, but is highly resource intensive for the healthcare [41]. During Mohs, repeated surgical removal of smaller pieces of the tumor is performed in stages without the patient leaving the clinic. Immediately after resection, the tissue is subject to frozen sectioning and is then assessed by a trained histopathologist regarding radicality and tumor growth direction to guide further removal of tissue if necessary. Since each excision stage requires a skilled surgeon and histopathologist that remove and process tissue as well as perform histological analysis, with 30–60 minutes expected duration per stage, total perioperative times can extend over several hours [42]. An automated method for preoperatively delineating superficial tumor margins and intraoperatively evaluating the resected sides for signs of tumor could help guide the surgeon to achieve clear margins more efficiently, thereby reducing the number of stages required and minimizing perioperative waiting times. Since HSI is relatively simple and uses harmless, non-ionizing radiation, it can be widely implemented in various other healthcare settings as well.

## 5. Summary

In conclusion, in this work we have demonstrated that combining a non-invasive imaging technique (HSI), together with ML methods enables preoperative delineation of skin tumors, providing surgical guidance to ensure complete tumor removal. The accuracy in predicted tumor sizes compared to histopathology is within the range considered surgically acceptable for tumor excision. With an accurate automated delineation, the risk for non-radical tumors and additional surgeries would be smaller, together with more healthy tissue being spared. This would reduce the total number of surgeries, and save both time, resources and improve quality of care for the patient. ML has great potential both for making the surgeries easier for the surgeon and making them more precise, resulting in better outcomes for the patient and their health. We emphasize that while the described workflow involves many steps where parameters are determined, the presented pipeline is a single algorithm that remains fixed for all examined samples but automatically adapts the hyperparameters to the data of the patient, which makes it both personalized and unsupervised, which has important implications for clinical translations.

## Data Availability

Data can be made available upon request.

## References

[1] (IARC) IAfRoC. Non-melanoma skin cancer fact sheet International Agency for Research on Cancer (Lyon, France)2024 [Available from: https://gco.iarc.who.int/media/globocan/factsheets/cancers/17-non-melanoma-skin-cancer-fact-sheet.pdf.

[2] Trust STHNF. Histology turnaround times 2025 [Available from: https://www.southtees.nhs.uk/services/pathology/cellular-pathology-2/histology-turnaround-times-2/.

[3] Rawlani R. Rawlani V. Qureshi H. A. et al., “Reducing margins of wide local excision in head and neck melanoma for function and cosmesis: 5-year local recurrence-free survival,” J. Surg. Oncol. 111(7), 795–799 (2015). 10.1002/jso.23886 25712156

[4] Lee K. S. Kim J. O. Kim N. G. et al., “A Comparison of the Local Flap and Skin Graft by Location of Face in Reconstruction after Resection of Facial Skin Cancer,” Arch Craniofac Surg 18(4), 255–260 (2017). 10.7181/acfs.2017.18.4.255 PMC5759659 29349050

[5] Răducu L. Avino A. Purnichescu Purtan R. et al., “Quality of Life in Patients with Surgically Removed Skin Tumors,” Medicina 56(2), 66 (2020). 10.3390/medicina56020066 PMC7074335 32050413

[6] Hult J Merdasa A Pekar-Lukacs A et al., “Comparison of photoacoustic imaging and histopathological examination in determining the dimensions of 52 human melanomas and nevi ex vivo,” Biomed. Opt. Express 12(7), 4097 (2021). 10.1364/BOE.425524 PMC8367235 34457401

[7] Stridh M. T. Hult J. Merdasa A. et al., “Photoacoustic imaging of periorbital skin cancer ex vivo: unique spectral signatures of malignant melanoma, basal, and squamous cell carcinoma,” Biomed Opt Express 13(1), 410 (2022). 10.1364/BOE.443699 PMC8803040 35154881

[8] Stridh M. Dahlstrand U. Naumovska M. et al., “Functional and molecular 3D mapping of angiosarcoma tumor using non-invasive laser speckle, hyperspectral, and photoacoustic imaging,” Orbit 43(4), 453–463 (2024). 10.1080/01676830.2024.2331718 38591750

[9] Dahlstrand U. Sheikh R. Malmsjö M., “Photoacoustic imaging for intraoperative micrographic control of the surgical margins of eyelid tumours,” Acta Ophthalmol. 98(2), e264–e5 (2020). 10.1111/aos.14245 31489789

[10] Parasca S. V. Calin M. A. Manea D. et al., “Hyperspectral imaging with machine learning for in vivo skin carcinoma margin assessment: a preliminary study,” Phys Eng Sci Med 47(3), 1141–1152 (2024). 10.1007/s13246-024-01435-8 PMC11408400 38771442

[11] Salmivuori M. Neittaanmäki N. Pölönen I. et al., “Hyperspectral imaging system in the delineation of Ill-defined basal cell carcinomas: a pilot study,” Acad Dermatol Venereol 33(1), 71–78 (2019). 10.1111/jdv.15102 29846972

[12] Naqvi M. Gilani S. Q. Syed T. et al., “Skin Cancer Detection Using Deep Learning—A Review,” Diagnostics 13(11), 1911 (2023). 10.3390/diagnostics13111911 PMC10252190 37296763

[13] Goyal M. Knackstedt T. Yan S. et al., “Artificial intelligence-based image classification methods for diagnosis of skin cancer: Challenges and opportunities,” Comput. Biol. Med. 127, 104065 (2020). 10.1016/j.compbiomed.2020.104065 PMC8290363 33246265

[14] Cullell-Dalmau M. Noé S. Otero-Viñas M. et al., “Convolutional neural network for skin lesion classification: understanding the fundamentals through hands-on learning,” Front. Med. 8, 644327 (2021). 10.3389/fmed.2021.644327 PMC7969634 33748163

[15] Merdasa A. Fracchia A. Stridh M. et al., “Advancing non-invasive melanoma diagnostics with deep learning and multispectral photoacoustic imaging,” Photoacoustics 45, 100743 (2025). 10.1016/j.pacs.2025.100743 PMC12272440 40686556

[16] Andersson E. Hult J. Troein C. et al., “Facilitating clinically relevant skin tumor diagnostics with spectroscopy-driven machine learning,” iScience 27(5), 109653 (2024). 10.1016/j.isci.2024.109653 PMC11053315 38680659

[17] Litjens G. Kooi T. Bejnordi B. E. et al., “A survey on deep learning in medical image analysis,” Med. Image Anal. 42, 60–88 (2017). 10.1016/j.media.2017.07.005 28778026

[18] Bishop CM., *Pattern Recognition and Machine Learning*. 1st ed. Springer: New York; 2006.

[19] Esteva A. Kuprel B. Novoa R. A. et al., “Dermatologist-level classification of skin cancer with deep neural networks,” Nature 542(7639), 115–118 (2017). 10.1038/nature21056 PMC8382232 28117445

[20] Nahhas A. F. Scarbrough C. A. Trotter S., “A Review of the Global Guidelines on Surgical Margins for Nonmelanoma Skin Cancers,” J Clin Aesthet Dermatol 10(4), 37–46 (2017). PMC5404779 28458773

[21] Merdasa A. Berggren J. Tenland K. et al., “Oxygen saturation mapping during reconstructive surgery of human forehead flaps with hyperspectral imaging and spectral unmixing,” Microvasc. Res. 150, 104573 (2023). 10.1016/j.mvr.2023.104573 37390964

[22] Lai H. Huang T. Lu B. et al., “Silhouette coefficient-based weighting k-means algorithm,” Neural Comput & Applic 37(5), 3061–3075 (2025). 10.1007/s00521-024-10706-0

[23] Siskou S. Pasquali P. Trakatelli M., “High Frequency Ultrasound of Basal Cell Carcinomas: Ultrasonographic Features and Histological Subtypes, a Retrospective Study of 100 Tumors,” J. Clin. Med. 12(12), 3893 (2023). 10.3390/jcm12123893 PMC10299541 37373588

[24] Hinz T. Ehler L. K. Hornung T. et al., “Preoperative characterization of basal cell carcinoma comparing tumour thickness measurement by optical coherence tomography, 20-MHz ultrasound and histopathology,” Acta Derm Venereol 92(2), 132–137 (2012). 10.2340/00015555-1231 22002855

[25] Pelosini L. Smith H. B. Schofield J. B. et al., “In vivo optical coherence tomography (OCT) in periocular basal cell carcinoma: correlations between in vivo OCT images and postoperative histology,” Br. J. Ophthalmol. 97(7), 890–894 (2013). 10.1136/bjophthalmol-2012-303043 23677987

[26] Barcaui E. O. Carvalho A. C. P. Valiante P. M. et al., “High-frequency (22-MHz) ultrasound for assessing the depth of basal cell carcinoma invasion,” Skin Res Technol 27(5), 676–681 (2021). 10.1111/srt.12999 33404160

[27] Auw-Haedrich C. Frick S. Boehringer D. et al., “Histologic safety margin in basal cell carcinoma of the eyelid: correlation with recurrence rate,” Ophthalmology 116(4), 802–806 (2009). 10.1016/j.ophtha.2008.11.012 19232734

[28] Sun M. T. Figueira E. Huilgol S. C. et al., “Minimum histological safety margins in periocular basal cell carcinoma,” Br. J. Ophthalmol. 98(5), 706.1 (2014). 10.1136/bjophthalmol-2013-304760 24457367

[29] Quazi S. J. Aslam N. Saleem H. et al., “Surgical Margin of Excision in Basal Cell Carcinoma: A Systematic Review of Literature,” Cureus 12(7), e9211 (2020). 10.7759/cureus.9211 PMC7430350 32821563

[30] Wang L. Shao A. Huang F. et al., “Deep learning-based semantic segmentation of non-melanocytic skin tumors in whole-slide histopathological images,” Exp Dermatol 32(6), 831–839 (2023). 10.1111/exd.14782 37017196

[31] Thomas S. M. Lefevre J. G. Baxter G. et al., “Non-melanoma skin cancer segmentation for histopathology dataset,” Data Brief 39, 107587 (2021). 10.1016/j.dib.2021.107587 PMC8627989 34877372

[32] Aloupogianni E. Ichimura T. Hamada M. et al., “Hyperspectral imaging for tumor segmentation on pigmented skin lesions,” J. Biomed. Opt. 27(10), 106007 (2022). 10.1117/1.JBO.27.10.106007 PMC9619132 36316301

[33] Bartoš V. Kullová M., “Basal cell carcinoma of the skin with mixed histomorphology: a comparative study,” Cesk Patol 52(4), 222 (2016). 27869450

[34] Geiger R. S. Cope D. Ip J. et al., ““Garbage in, garbage out” revisited: What do machine learning application papers report about human-labeled training data?” Quantitative Science Studies 2(3), 795–827 (2021). 10.1162/qss_a_00144

[35] Fernández-Delgado M. Cernadas E. Barro S. et al., “Do we need hundreds of classifiers to solve real world classification problems?” J. Mach. Learn. Res. 15(1), 3133 (2014).

[36] Gregory N. Mulvaney M. Pattison T. et al., “Shrinkage of skin excision specimens and downcoding,” Arch. Dermatol. 139(4), 542 (2003). 10.1001/archderm.139.4.542 12707111

[37] Gardner E. S. Sumner W. T. Cook J. L., “Predictable tissue shrinkage during frozen section histopathologic processing for Mohs micrographic surgery,” Dermatol. Surg. 27(9), 813–818 (2001). 10.1046/j.1524-4725.2001.01017.x 11553170

[38] Golomb F. M. Doyle J. P. Grin C. M. et al., “Determination of preexcision surgical margins of melanomas from fixed-tissue specimens,” Plast Reconstr Surg 88(5), 804–809 (1991). 10.1097/00006534-199111000-00010 1924566

[39] Kerns M. J. Darst M. A. Olsen T. G. et al., “Shrinkage of cutaneous specimens: formalin or other factors involved?” J. Cutaneous Pathol. 35(12), 1093–1096 (2008). 10.1111/j.1600-0560.2007.00943.x 18544064

[40] Dauendorffer J. N. Bastuji-Garin S. Guéro S. et al., “Shrinkage of skin excision specimens: formalin fixation is not the culprit,” Br. J. Dermatol. 160(4), 810–814 (2009). 10.1111/j.1365-2133.2008.08994.x 19183182

[41] Gnanendran S. Aghajani M. Elhindi J. et al., “Mohs Micrographic Surgery for Basal Cell Carcinoma: Predictive Factors for Increased Stages and Relative Defect Sizes,” Australas. J. Dermatol. 66(5), 258–267 (2025). 10.1111/ajd.14535 40464309

[42] Wang W. Lee J. Qin S. et al., “Indications and Outcomes of Mohs Micrographic Surgery Using a Multidisciplinary Approach: A Decade of Experience,” Dermatol. Surg. 47(1), 10–15 (2021). 10.1097/DSS.0000000000002467 32541342

